# miR-145, miR-92a and miR-375 Show Differential Expression in Serum from Patients with Diabetic Retinopathies

**DOI:** 10.3390/diagnostics12102275

**Published:** 2022-09-21

**Authors:** Adriana Solis-Vivanco, Mónica Santamaría-Olmedo, Dalila Rodríguez-Juárez, Margarita Valdés-Flores, Carlos González-Castor, Rafael Velázquez-Cruz, Eric Ramírez-Salazar, Ana Cristina García-Ulloa, Alberto Hidalgo-Bravo

**Affiliations:** 1 Department of Ophthalmology, National Institute of Rehabilitation (INR), Calzada Mexico-Xochimilco 289, Arenal de Guadalupe, Mexico City 14389, Mexico; 2 Department of Genomic Medicine, National Institute of Rehabilitation (INR), Calzada Mexico-Xochimilco 289, Arenal de Guadalupe, Mexico City 14389, Mexico; 3 National Institute of Genomic Medicine (INMEGEN), Periférico Sur 4809, Arenal Tepepan, Mexico City 14610, Mexico; 4 Centro de Atención Integral del Paciente con Diabetes, National Institute of Medical Sciences and Nutrition (INCMNSZ), Vasco de Quiroga 15, Belisario Domínguez Secc 16, Tlalpan, Mexico City 14080, Mexico

**Keywords:** diabetic retinopathies, proliferative diabetic retinopathy, diabetic macular edema, microRNAs

## Abstract

Diabetic retinopathies are important disabling conditions. Micro-RNAs (miRNAs) are regulators of gene expression and diseases can change their expression. Our aim was to analyze the expression of miRNAs in serum and vitreous samples from patients with diabetic retinopathies. The following groups and number of individuals were included: proliferative diabetic retinopathy (PDR) (*n* = 16), diabetic macular edema (DME) (*n* = 17), and idiopathic epiretinal membrane (IEM) as non-diabetic controls (*n* = 23). The initial miRNA expression was explored using TaqMan low-density arrays (TLDAs) with subsequent validation through a quantitative polymerase chain reaction (qPCR). Target genes were identified through bioinformatic tools for enrichment analysis. The TLDAs revealed the following miRNAs with differential expression in terms of PDR vs. IEM: miR-320a-3p, miR-92a-3p, and miR-375-3p in the serum, with miR-541-5p and miR-223-5p in the vitreous samples. DME vs IEM: miR-486-5p, miR-145-5p, miR-197-3p, and miR-125b-5p in the serum, and miR-212-3p in vitreous samples. PDR vs. DME: miR-486-5p, miR-100-5p, miR-328-3p, miR-660-5p, and miR-145 in the serum and none in the vitreous samples. Validation was confirmed only for miR-145, miR-92a, and miR-375 in the serum. The relevant enriched pathways for these three validated miRNAs, miR-145, miR-92a, and miR-375 were the vascular endothelial growth factor and its receptor, hepatocyte growth factor receptor, epidermal growth factor, focal adhesion, and phosphoinositide 3-kinase. Our results support the involvement of miRNAs in the pathophysiology of diabetic retinopathies and reinforce their potential as biomarkers or therapeutic resources.

## 1. Introduction

Diabetes mellitus (DM) is one of the most prevalent diseases worldwide. In Mexico, visual disability occupies the second-highest place among diabetic complications, affecting 40% of patients [1]. Proliferative diabetic retinopathy (PDR) and diabetic macular edema (DME) represent the main ophthalmic complications. PDR progresses to retinal detachment and neovascular glaucoma, while DME implies central vision impairment. Currently, the diagnosis of PDR and DME is made under observation with a slit lamp. Despite the progression in treatment strategies, the pathophysiology of ophthalmologic complications related to DM is not fully understood.

In recent years, microRNAs (miRNAs) have emerged as important regulators of gene expression in approximately 60% of human genes. miRNAs are non-coding RNA molecules of 21 to 24 nucleotides that have the ability to modulate the availability of a protein. They are involved in the posttranscriptional regulation of gene expression through mRNA degradation or translation repression [2,3]. miRNAs participate in most of the biological processes, both normal and pathological [4]. In addition, miRNAs can be detected in several bodily fluids, which makes them attractive potential biological markers [5,6,7]. In the last decade, some studies have identified miRNAs that are differentially expressed in patients with certain eye diseases [7,8,9]. The evidence has demonstrated the differential expression of miRNAs in the serum, vitreous, and other eye tissues in relation to a pathological condition affecting different structures in the eye [10]. Focusing on diabetic complications affecting the retina, some miRNAs have been found with differential expression in the vitreous and serum samples [11]. Qing et al. proposed a special serological sieve of microRNAs that can serve as a “signature” of PDR, which could precede early diagnosis [12]. The identification of these miRNAs might provide additional knowledge regarding the genes and metabolic pathways undergoing modification during the progression of the disease. The ultimate aim is the discovery of potential disease markers and therapeutic targets. Their dysregulation in metabolic diseases underlines their potential as therapeutic targets [13]. At the same time, they can help us to understand the disparity in severity and the development of complications between individuals. Identifying the miRNAs with differential expression in diabetic patients would represent a link in the chain of knowledge that is required for the creation of new therapeutic strategies. Cumulative evidence supports the theory that miRNAs could serve as biomarkers for supporting diagnosis and follow-up. The aim of this study was to analyze the expression of miRNAs in the serum and vitreous samples from diabetic patients with established retinal microangiopathy.

## 2. Materials and Methods

### 2.1. Study Population

Patients attending the ophthalmology department at the National Institute of Rehabilitation in Mexico City from 2018 to 2019 were invited to participate. We recruited 16 type-2 diabetic patients with PDR who showed high-risk characteristics without DME, 17 type-2 diabetic patients with severe diffuse DME, and 23 non-diabetic patients with idiopathic epiretinal membrane (IEM). Individuals with IEM were chosen as controls because it is a pathology not associated with a metabolic disorder and that requires vitrectomy as treatment since we cannot obtain vitreous samples from healthy patients without justification. All participants were older than 35 years old, with a recommendation for pars plana vitrectomy. All individuals were of Mexican origin, with at least two generations of Mexican ancestors. Exclusion criteria included those patients who had had anti-VEGF treatment six months before. Patients who had vitreous hemorrhage at the time of surgery were also excluded, to avoid contamination of the vitreous content of miRNAs. In the case of the RDP group, we were especially careful in selecting patients who required posterior vitrectomy because of advanced diabetic retinopathy (tractional retinal detachment), without vitreous hemorrhage. In the case of the DME group, the indication for vitrectomy was to perform a hyaloidectomy and internal limiting membrane peeling to improve the macular thickness. None of these patients had proliferative diabetic retinopathy; therefore, there was no vitreous hemorrhage. All procedures were performed in accordance with the principles stated in the Declaration of Helsinki. All protocol procedures were approved by the Institutional Ethics Committee (approval number 19/16) and an informed consent form was signed by all participants during an interview with A.S.V. or D.R.J.

### 2.2. Vitreous Sample

Prior to starting the vitrectomy and before opening the infusion of physiological solution, 1 mL of vitreous was cut and aspirated with the vitrector. The vitreous sample was aspirated with a sterile technique using a 1 mL syringe to extract it from the vitrector equipment hoses. The vitreous sample was placed in a sterile microtube for centrifugation and storage at −80 °C until use.

### 2.3. RNA Isolation

Three mL of whole blood were obtained from each participant using BD Vacutainer blood collection tubes (Becton, Dickinson, and Company, Franklin Lakes, NJ, USA), while serum was separated by centrifugation at 5000 rpm for 10 min. Total RNA was extracted from 200 μL of serum and 200 μL of vitreous samples using the miRNeasy Serum/Plasma Kit (QIAGEN, Germantown, MD, USA, Cat. No. 217184) according to the manufacturer’s instructions. RNA concentration and purity were analyzed using a BioDrop spectrophotometer (Biodrop, Cambridge, United Kingdom). RNA pools were prepared for each study group containing equal amounts of total RNA. Each pool contained total RNA from eight individuals; therefore, we generated 3 pools of total RNA derived from the serum and 3 pools of total RNA derived from the vitreous samples. The same individuals integrated the pools of serum-derived and vitreous-derived total RNA.

### 2.4. microRNA Profiling

In each pool of RNA, quantitative global profiling of serum and vitreous miRNAs was performed using the low-density TaqMan arrays (TLDAs) Megaplex (Applied Biosystems, Foster City, CA, USA, Cat. No. 4444913) which includes panels A and B. Panel A contains 384 TaqMan microRNA assays, enabling the simultaneous quantification of 377 human mature miRNAs, in addition to 4 endogenous controls. Panel B contains 290 human mature miRNA assays, in addition to 7 endogenous controls. Differentially expressed miRNAs were identified through the Expression Suite Software (Applied Biosystems). Both panels were prepared following the manufacturer’s recommendations; briefly, 3 μL of total RNA from each pool were reverse-transcribed with the Megaplex RT primer (Applied Biosystems, California, USA, Cat. No. 4444913). The RT products were then pre-amplified using Megaplex PreAmp Primers and the TaqMan PreAmp Master Mix. The cDNA was diluted to 1:8 with distilled water and subsequently distributed into the 384 wells via centrifugation. The real-time PCR cycling parameters were set according to the manufacturer’s recommendations using a QuantStudio 7 instrument (Applied Biosystems, Foster City, CA, USA). Expression data from the cards were analyzed using the Expression Suite software (Applied Biosystems, Foster City, CA, USA), considering for expression analysis only those miRNAs with a raw Cq value below 35. The fold change was estimated using the 2^−ΔΔCt^ method. The normalization factor was represented by the global mean expression value of all miRNAs.

### 2.5. qPCR Validation Analysis

The miRNAs with the most significant differential expression and published evidence regarding their potential involvement in diabetic microangiopathies were selected for further validation. Validation was carried out in the three groups of the entire population study through qPCR. For validation, 3 µL of total RNA at a concentration of 10 ng/μL was used as the template for reverse transcription, using the TaqMan MicroRNA Reverse Transcription Kit (Applied Biosystems, Foster City, CA, USA, Cat. No. 4366596). We scaled the contents of the retro-transcription reactions to obtain enough cDNA for analyzing all the genes of interest. The final volume of the RT reactions was 30 µL. Each reaction was diluted to a final volume of 100 µL with RNase-free water. Afterward, 5 µL were used as the template for qPCR reactions to analyze the expression of the selected miRNAs, using predesigned TaqMan microRNA assays (ThermoFisher, Waltham, MA, USA): hsa-miR-145-5p (Cat. No. 002278), hsa-miR-92a-3p (Cat. No. 000431), hsa-miR-375-3p (Cat. No. 000564), hsa-miR-486-5p (Cat. No. 001278), hsa-miR-212-3p (Cat. No. 000515) and hsa-miR-223-5p (Cat. No. 002098). qPCRs were performed using a QuantStudio 7 instrument (Applied Biosystems, Foster City, CA, USA). The expression levels were normalized using RNU6 as the reference gene. All the qPCR reactions were performed in triplicate. The expression fold change of all miRNAs was determined using the 2^−ΔΔCt^ method.

### 2.6. Statistical Analysis

Demographic features were analyzed using the IBM SPSS 15 statistics software. Student’s *t*-test was used for comparing two quantitative variables, a one-way ANOVA test when comparing the three groups, and a chi-square test when comparing the proportions of categorical variables. Expression Suite Software Version 1.3 (Life Technologies, Carlsbad, CA, USA) was used to assess the differential expression of miRNAs between the study groups for the TLDAs experiments. miRNA with a fold change of ≥1.5 or ≤0.7 and a *p*-value of <0.05 were considered for further validation. The expression levels of validated miRNAs were compared through a Kruskal–Wallis test with paired comparisons. A correlation analysis using Spearman’s test was conducted to investigate if the levels of the validated miRNAs correlated with the values of glycemia, best corrected visual acuity, and the number of years since the diagnosis of T2DM.

### 2.7. Target Prediction and Gene Enrichment Analysis

Target genes for those miRNAs for which differential expression was validated through qPCR were obtained using the miRNet database [14]. These target genes were used as input for pathway enrichment analysis, using WebGestalt (a web-based gene set analysis toolkit) [15]. Parameters for the analysis were over-representation analysis (ORA) being the method used, with the Kyoto Encyclopedia of Genes and Genomes (KEGG) as the functional database selected. Interaction networks between miRNAs and target genes were built on miRNet. The interaction of pathways was depicted using the enrichment map app for Cytoscape software [16,17].

## 3. Results

### 3.1. Clinical Data

Samples were successfully collected from 17 patients with DME, 16 patients with PDR, and 23 patients with IEM. The mean ages were significantly different between groups (*p* < 0.001). The predominant sex in the three groups was female, although the proportion difference is not significant (*p* = 0.614). The mean of the years since DM diagnosis was longer in the DME group compared to the PDR group (*p* = 0.003). The mean best corrected visual acuity (BCVA) was lower in the DME group (log-transformed in Table 1) without significance vs. controls but, when comparing both diabetic groups, patients with DME have significantly less vision than PDR (*p* < 0.001). About 27% of diabetic patients had been treated with an anti-VEGF more than 12 months prior to their participation in the study. The preoperative mean of glycemia is higher in the DME group (*p* = 0.010). Only one patient in the PDR group and two in the DME group had diabetic nephropathy. No other DM-related complications were reported in the population study. Regarding the control group comprising individuals with IEM, DM was ruled out in all of them. The mean age of the IEM group was significantly higher than the DM patients; this is related to the usual age of presentation of IEM. The average visual acuity in this group was 0.7 in logMAR, which is roughly 20/100 on Snellen’s chart (Table 1).

### 3.2. TLDAs miRNA Expression Analysis

The expression analysis, through TLDAs, of each study group was performed by duplicate. When we compared expression profiles of patients with PDR against IEM, three miRNAs showed differential expression in the serum, hsa-miR-320a-3p, hsa-miR-92a-3p, and hsa-miR-375-3p, and two did so in the vitreous samples, hsa-miR-541-5p and hsa-miR-223-5p. When the comparison was between patients with DME against IEM, four miRNAs had significant differential expression; in the serum, these were hsa-miR-486-5p, hsa-miR-197-3p, and hsa-miR-125b-5p, while one in the vitreous samples, it was hsa-miR-212-3p. In the comparison between both groups of diabetic patients (PDR vs. DME, considering DME as the reference), five miRNAs were differentially expressed in the serum, hsa-miR-486-3p, hsa-miR-100-5p, hsa-miR-328-3p, hsa-660-5p, and hsa-145-5p. In the vitreous samples, the comparison between these two groups did not show any miRNA being differentially expressed. Table 2 summarizes these findings.

### 3.3. Target Genes Identification and Enrichment Analysis

**Figure 1:**
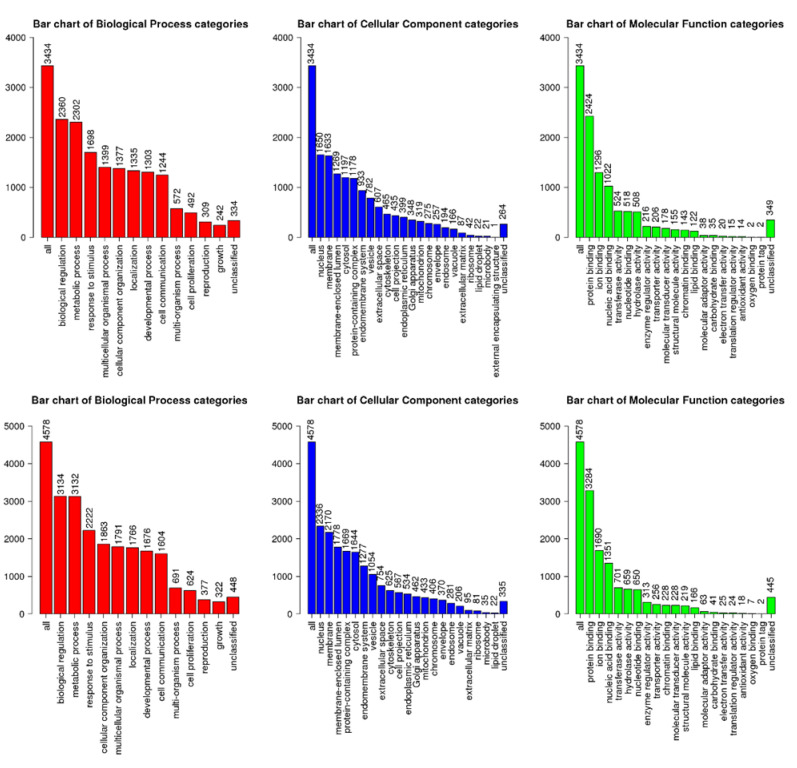
Gene ontology analysis of the target genes under the regulation of the miRNAs, showing differential expression in the TLDAs assay. Top comparison of proliferative diabetic retinopathy vs. idiopathic epiretinal membrane. Bottom: comparison of diabetic macular edema vs. idiopathic epiretinal membrane. Categories of biological processes in red, categories of cellular component in blue and categories of molecular function in green.

A total of 14 miRNAs showed differential expression in the TLDAs assay. To further identify the enriched functions under the regulation of the differentially expressed miRNAs, we used the target genes as the input for enrichment analysis. The analysis using miRNet revealed 1911 annotated target genes, with miRNA response elements in their 3’UTR region, for the five miRNAs that were differentially expressed in the comparison of PDR vs. IEM. For the four miRNAs with differential expression when comparing DME vs. IEM, 1417 annotated target genes were identified. These target genes were used as input for enrichment analysis. Table 3 depicts the enriched pathways from each comparison. For both comparisons, gene ontology analysis showed that the majority of the target genes participate in biological regulation, are expressed in the nucleus, and have a function related to protein binding (Figure 1).

### 3.4. qPCR Validation of miRNAs

**Figure 2:**
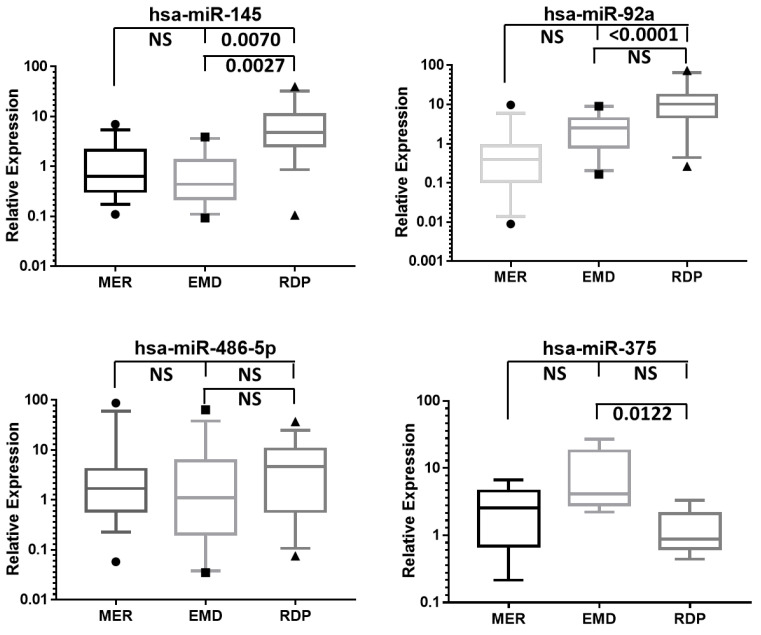
Relative expression of the four validated miRNAs in the serum. Four miRNAs were validated in all participants of the three study groups. *p*-values of the pairwise comparisons are shown (NS, not a significant *p*-value). The graph presents medians with an interquartile range. IEM, idiopathic epiretinal membrane. DME, diabetic macular edema. PDR, proliferative diabetic retinopathy.

We conducted a literature review focused on those miRNAs showing differential expression in the TLDAs analysis. Based on the previous evidence reported, four miRNAs in the serum, hsa-miR-145, hsa-miR-92a-3p, hsa-miR-486-5p, and hsa-miR-375-3p, and two in the vitreous samples, hsa-miR-223-5p and hsa-miR-212-3p, were selected for further validation through qPCR. Validation assays were carried out in all the individuals from each study group. The analysis of the four miRNAs in the serum validated the differential expression in three of them, hsa-miR-375-3p, hsa-miR-92a-3p, and hsa-miR-145, between the study groups. Further analysis revealed significant differences between all the possible pairs of groups (Figure 2).

Interestingly, there were no significant differences between the IEM and DME groups. On the other hand, the expression of hsa-miR-145 and hsa-miR-92a-3p was significantly higher in the PDR group compared to the IEM group. The only miRNA with differential expression between the two groups of diabetic individuals was hsa-miR-375-3p, which showed higher expression in the DME group compared to the PDR group. We also performed qPCR validation in two miRNAs from the vitreous samples, hsa-miR-212-3p and hsa-miR-223-5p. These two miRNAs could not be detected in most of the samples of the three groups, even when the reference gene was amplified correctly. Therefore, we could not validate these two miRNAs. The correlation analysis between the three validated miRNAs and the levels of glycemia, the number of years since the diagnosis of T2DM, and the BCVA did not reveal statistically significant correlations (data not shown).

### 3.5. Enrichment Analysis

**Figure 3:**
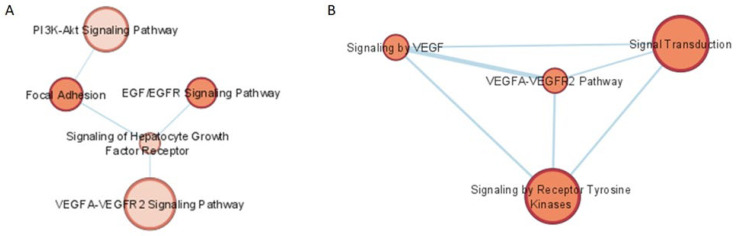
Enriched pathways interaction. The target genes of the three validated miRNAs with differential expression in the serum were used as input for enrichment analysis in the KEGG, Reactome, and Wikipathways databases. The VEGF-related pathways were enriched in Wikipathways (**A**) and Reactome (**B**).

The target genes of the three validated miRNAs were obtained using the miRNet database; a total of 1326 targets were recognized. These genes were used as input for functional enrichment analysis in gProfiler for searching in the KEGG, Reactome, and Wikipathways databases. Afterward, the pathways’ interaction networks were built using the Enrichment Map app for Cytoscape. Reactome and Wikipathways revealed enrichment of the VEGF-related pathways and the closest interacting pathways were also identified (Figure 3). The focal adhesion pathway also showed enrichment in two databases, Wikipathways and KEGG.

## 4. Discussion

Genetic studies are powerful tools for dissecting the molecular mechanisms underlying diabetic microangiopathies. A variety of genes have been reported to be involved in the development and progression of diabetic retinopathy [3]. Heritability has been estimated to be between 27% to 52% for these disorders [18]. The evidence demonstrating the dysregulation of miRNAs involved in DM-related metabolic pathways is constantly growing [19], and the observation that some miRNAs share mechanisms of action with multi-system diabetic microangiopathy is interesting [4]. The importance of miRNAs in regulating angiogenesis has been revealed by in vitro and in vivo studies [20].

Among the miRNAs involved in the modulation of crucial pathways for glucose metabolism, miR-375 is highly expressed in pancreatic β-cells, and is able to directly reduce insulin secretion [21,22]. We observed the upregulation of miR-375; levels of this miRNA have been found in relation to the damage at the zonula adherens and zonula occludens. The zonula occludens is closely related to the breakdown of the blood–retinal barrier. In addition, miR-375 has shown differential expression in retinal tissue derived from diabetic rats [23]. It also can regulate rat pulmonary microvascular endothelial cell activity during hypoxia by targeting Notch1 [24]. Notch1 represents a crucial pathway regulating angiogenesis in diabetic retinopathy [25]. The TLDA assay found the downregulation of miR-320 in the serum of patients with PDR, although it was not successfully validated by qPCR. In diabetic rats, miR-320 suppresses the glucose-induced increase in VEGF [26]. miR-320 has been proposed as a potential predictor of retinopathy progression in patients with type 1 DM [27]. In addition, miR-320 has been related to endothelial damage [28], targeting the inflammatory metabolic pathways. Another relevant miRNA that is downregulated in patients with PDR was hsa-miR-92a-3p; it has been described as an inhibitor of oxidative stress [20] and as a predictor of acute coronary syndrome in diabetic patients [29]. miR-92a has also been recognized as part of the endogenous miRNAs regulating the genes involved in hypertension in endothelial cells [30]. Silencing of miR-92a reduces oxidative stress and injury in diabetic nephropathy [31]. Bonauer et al. showed that hsa-miR-92a controls the growth of new blood vessels and its overexpression in endothelial cells blocks angiogenesis in vitro and in vivo [32]. miR-92a inhibits angiogenesis by targeting VEGFA and integrin subunit alpha5 [33]. miR-92a could also be part of the molecular mechanisms promoting retina neovascularization in patients with PDR. Although miR-92a seems a potential therapeutic resource, it is important to consider that even when restoring its expression for inhibiting vascularization in the retina there could be off-target effects on other tissues. miR-145 has also been associated with a senescent phenotype of smooth muscle cells (SMC) derived from patients with T2DM. This senescent phenotype can lead to DNA damage, with further vascular dysfunction [34]. Therefore, miR-145 has the potential to become a clinically useful biomarker of vascular damage.

For the initial enrichment analysis, we considered the target genes of 14 miRNAs with differential expression through TLDAs. The metabolic pathways that were enriched were related to apoptosis, focal adhesion, adherens, and tight junction pathways. All these pathways are involved in pericyte loss, which is a fundamental event for the development of DR [27,34,35]. Afterward, we used the target genes of the three validated miRNAs as input for enrichment analysis. The enriched pathways were hepatocyte growth factor (HGF) and its receptor (HGFR), epidermal growth factor and its receptor (EGF and EGFR), P13K-AKt, and the tyrosine kinases receptor (RTKs) and vascular endothelial growth factor (VEGFA and VEGFR). HGF is a multifunction cytokine that plays an important role in pancreatic physiology. In terms of retinal diabetic microangiopathies, HGF seems to be involved in pericyte survival by increasing the AKT signaling pathway, leading to the strengthening of the endothelial tight junction [36]. On the other hand, the aqueous levels of HGF correlate with macular edema severity; it was increased in the cells and macrophages associated with retinal neovascularization in the murine model of an ischemic retina [37]. Several RTKs have been involved in angiogenesis, such as the EGF and its receptor, EGFR. The EGFR family of RTKs have been found to be involved in multisystemic diabetic angiopathies [38,39,40], even though EGF has been proposed as a biomarker for DR [41]. Regarding the phosphoinositide 3-kinase (PI3K)/Akt pathway, Han et al. have shown that normal vitreous has the ability to promote the growth of human pigment retinal epithelial and endothelial cells through this pathway [42]. The phosphoinositide 3-kinase (PI3K)/Akt pathway has also been linked to hyperglycemia-induced migration, proliferation, and the angiogenesis dysfunction of endothelial cells in diabetic patients and represents a growth-regulating cellular signaling pathway. They also showed that vitreous increases its own proliferation, migration, and tube formation via EGFR in human umbilical vein endothelial cells (HUVECs). On the other hand, the finding that the suppression of vitreous-induced Akt activation, cell proliferation, and migration by blocking the EGFR in cell cultures exalts the importance of the vitreous in the physiopathology of angiogenesis-related ophthalmic diseases [43]. 

Crucial elements for PDR development are the permeability of the blood-retinal barrier (BRB) and the importance of pericytes and endothelial cells focal adhesions (EC-FAs) as principal structural components. Focal adhesions (FAs) are composed of a high density of proteins and provide dynamic links between the extracellular matrix and intracellular cytoskeleton. Focal adhesion kinase (FAK) or vinculin is a non-receptor protein tyrosine kinase. It is principally located in the FAs and is a key molecule involved in the control of pericyte migration; this control is loss in the diabetic retina. Pericyte migration promotes increased vascular permeability and BRB leakage, which represent an early feature of PDR pathology [44,45].

Interestingly, some of the miRNAs that are most reported in the literature associated with PDR or DME did not show differential expression in our results. However, others fully coincided, and we also found miRNAs with significant expression, described for the first time in association with retinal microangiopathy. Our results add to the evidence about the role of miRNAs in the pathogenesis of DM complications affecting the retina. Nevertheless, more research is necessary to achieve a better understanding of these conditions and define the most suitable miRNAs for clinical use.

We were unable to validate by qPCR those miRNAs with differential expression in vitreous samples observed through the TLDAs assays. One possible explanation is the relatively small number of individuals in each group and the low abundance of miRNA present in the vitreous samples. Further validation may require a larger sample.

This study has some limitations; first, the sample size could seem relatively small. Nevertheless, the validated miRNAs regulate the target genes participating in pathways related to the physiopathology of diabetic microangiopathies. Second, we were not able to validate the miRNAs present in the vitreous samples; one possible reason could be related to the sample size, where a larger sample size could overcome the lack of detection of these miRNAs in some samples. Nevertheless, our study adds to the evidence regarding the potential role of miRNAs in ophthalmic complications in T2DM patients. Based on these and the evidence discussed above, it is tempting to assign a clinical use to the validated miRNAs. miR-375 has the potential to be a useful biomarker of disease initiation, while miR-92a has the potential to become a promising therapeutic resource, considering its effects on angiogenesis. Finally, miR-145 has the potential to be a biomarker of vascular dysfunction. All these hypotheses need to be evaluated, with a proper study design. 

## Figures and Tables

**Table 1:** Clinical and demographical features of study groups.

Study Groups				
	PDR (*n* = 16)	DME (*n* = 17)	IEM (*n* = 23)	*p*-Value
**Age, mean (SD)**	55.65 (10.74)	64.25 (9.48)	77.69 (8.42)	^a^*p* < 0.001
**Sex**				
**Male *n* (%)**	4 (25)	7 (41)	8 (35)	^b^*p* = 0.614
**Female *n* (%)**	12 (75)	10 (59)	15 (65)	
**Years from DM diagnosis, mean (SD)**	16.3 (8.51)	21.26 (10.56)	*n*/A	^c^*p* = 0.003
BCVA, mean logMAR * (SD)	1.0 (0.35)	1.5 (0.27)	0.7 (0.25)	^a^*p* < 0.001 ^d^ *p* = 0.173 ^e^ *p* = 0.064 ^f^ *p* < 0.001
**Previous Anti-VEGF treatment *n* (%)**	3 (18.75)	6 (35.29)	*n*/A	^c^*p* = 0.286
**Glycemia (mg/dL), mean (SD)**	114.25 (45.15)	121.16 (41.95)	96.08 (16.66)	^a^*p* = 0.071 ^d^ *p* = 0.0032 ^e^ *p* = 0.0022 ^f^ *p* = 0.010
**Diabetes mellitus-related complications**				
**Diabetic Nephropathy *n* (%)**	1 (6.25%)	2 (11.76%)	*n*/A	^f^*p* = 0.581

PDR: proliferative diabetic retinopathy, DME: diabetic macular edema, IEM: idiopathic epiretinal membrane, DM: diabetes mellitus, BCVA: best corrected visual acuity, SD: standard deviation, VEGF, vascular endothelial growth factor. Age is expressed in years. ^a^ *p*-value derived from ANOVA comparing the three groups. ^b^ *p*-value for proportions comparison through a chi-square test. ^c^ *p*-value obtained through a *t*-test comparing PDR vs. DME groups. ^d^ *p*-value from PDR vs. IEM comparison. ^e^ *p*-value from DME vs. IEM comparison. ^f^ *p*-value from PDR vs. DME comparison. * logMAR (logarithm of the minimum angle of resolution, it is a base-10 logarithm to enable a more accurate estimate of visual acuity).

**Table 2:** Differentially expressed microRNAs in the serum and vitreous samples, obtained through TLDAs.

	PDR vs. IEM			DME vs. IEM			PDR vs. DME		
	hsa-miR	*p*-Value	FC	hsa-miR	*p*-Value	FC	hsa-miR	*p*-Value	FC
**Serum**	**375-3p**	0.004	2.84	**486-5p**	0.018	0.382	486-3p	0.018	0.382
	320a-3p	0.021	0.687	197-3p	0.014	0.533	100-5p	0.033	0.41
	**92a-3p**	0.0029	0.934	125b-5p	0.019	0.112	328-3p	0.038	0.481
							660-5p	0.039	2.719
							**145-5p**	**0.010**	**2.72**
**Vitreous**	**223-5p**	0.016	0.360	**212-3p**	0.005	6.412			
	541-5p	0.016	0.390						

In bold: miRNAs selected for validation through qPCR. In the comparison of PDR vs. DME, DME was considered the group of reference. PDR: proliferative diabetic retinopathy, DME: diabetic macular edema, IEM: idiopathic epiretinal membrane, FC: fold change.

**Table 3:** Enriched pathways based on the target genes under the regulation of miRNAs with differential expression, found through TLDAs analysis.

Description	Ratio	*p*-Value	FDR
**PDR vs. IEM**			
Cell cycle	2.087	1.008 × 10^−11^	3.287 × 10^−9^
Adherens junction	2.272	1.949 × 10^−9^	1.059 × 10^−7^
AGE-RAGE signaling pathway in diabetic complications	1.883	5.621 × 10^−7^	1.078 × 10^−5^
EGFR tyrosine kinase inhibitor resistance	1.926	2.952 × 10^−6^	4.375 × 10^−5^
Focal adhesion	1.530	9.972 × 10^−6^	1.161 × 10^−4^
ErbB signaling pathway	1.835	1.055 × 10^−5^	1.186 × 10^−4^
TGF-beta signaling pathway	1.767	5.141 × 10^−5^	5.079 × 10^−4^
AMPK signaling pathway	1.617	7.505 × 10^−5^	6.796 × 10^−4^
HIF-1 signaling pathway	1.636	1.962 × 10^−4^	1.330 × 10^−3^
Tight junction	1.477	1.999 × 10^−4^	1.330 × 10^−3^
**DME vs. IEM**			
Cell cycle	2.5656	1.421 × 10^−14^	2.316 × 10^−12^
TGF-beta signaling pathway	2.3213	6.262 × 10^−8^	2.807 × 10^−6^
AGE-RAGE signaling pathway in diabetic complications	2.1251	3.783 × 10^−7^	9.487 × 10^−6^
EGFR tyrosine kinase inhibitor resistance	2.2084	1.295 × 10^−6^	2.380 × 10^−5^
HIF-1 signaling pathway	2.0525	1.533 × 10^−6^	2.380 × 10^−5^
AMPK signaling pathway	1.7959	4.239 × 10^−5^	3.735 × 10^−4^
Endocrine resistance	1.8326	1.138 × 10^−4^	8.631 × 10^−4^
ErbB signaling pathway	1.8714	1.781 × 10^−4^	1.290 × 10^−3^
Adherens junction	1.9242	2.713 × 10^−4^	1.805 × 10^−3^
TNF signaling pathway	1.726	2.987 × 10^−4^	0.0019

FDR, false discovery rate. PDR, proliferative diabetic retinopathy. IEM, idiopathic epiretinal membrane. DME, diabetic macular edema.

## Data Availability

Data are available under reasonable request to the corresponding author.
